# Comparative analysis of adult distal radius fracture reduction in the emergency room: fluoroscopy-guided vs. non-fluoroscopy-guided approaches

**DOI:** 10.1007/s00402-025-06132-z

**Published:** 2025-11-27

**Authors:** Shay Ribenzaft, Ran Atzmon, Tomer Rubin, Samuel Cohen, Shai Shemesh

**Affiliations:** 1 Department of Orthopedic Surgery, Rabin Medical Center, Petah Tikva, Israel; 2 Gray Faculty of Medicine, Tel Aviv University, Tel Aviv, Israel; 3 Department of Orthopaedic Surgery, Assuta Ashdod Medical Center, Ashdod, Israel; 4 Faculty of Health Sciences, Ben-Gurion University of the Negev, Beersheba, Israel

**Keywords:** Distal radius fracture, Guided reduction, Fluoroscopy, Emergency room procedures, Radiographic outcomes

## Abstract

**Introduction:**

Distal radius fractures are a common injury, typically treated with closed reduction and plaster casting. Fluoroscopy is frequently used during the reduction process to ensure accurate alignment. However, the necessity of fluoroscopy in achieving optimal radiographic outcomes remains uncertain. While fluoroscopy is considered the gold standard in many settings, concerns about radiation exposure and the cost of its routine use, especially in resource-limited environments, have sparked interest in non-fluoroscopy-guided methods.

**Research objectives:**

The primary objective of this study is to assess the radiographic outcomes of distal radius fractures after reduction and casting in plaster, comparing cases treated without fluoroscopy to those treated with fluoroscopy during the procedure. Secondary objectives include comparing the groups based on the percentage of fractures meeting conventional indications for surgery or non-surgical treatment after reduction, the average time spent in the emergency department (ED), and follow-up visits to the ED within the subsequent week due to plaster complications. It was hypothesized that fluoroscopy would have a minimal impact on the final X-ray results following closed reduction and casting.

**Methods:**

This retrospective study reviewed the records of patients who visited the ED between 2015 and 2021 with distal radius fractures and received initial treatment involving reduction and casting. Patients were divided into two groups: one treated with fluoroscopy-guided closed reduction and the other with non-fluoroscopy-guided techniques. The study compared radiographic and clinical outcomes, including the need for surgery, complications, and return visits to the ED due to plaster-related issues. Statistical analysis was performed to identify significant differences between the two groups.

**Results:**

Of the 85 participants included in the study who underwent reduction and casting, 45 were treated with fluoroscopic guidance while 40 were treated without fluoroscopy. No significant differences were found between the two groups in the radiographic outcomes, including radius length, inclination, posterior angulation, and step-off, between the fluoroscopy-guided and non-fluoroscopy-guided groups. Furthermore, no difference was found in the percentage of fractures requiring surgery or in the rate of return visits to the ED due to plaster complications. Although there were some minor differences in posterior angulation and radial height between the groups, these differences did not translate into meaningful clinical benefits, such as improved functional recovery or reduced need for surgery.

**Conclusions:**

The use of fluoroscopy did not demonstrate an improvement in radiographic outcomes for conventional measures of closed reduction and casting in distal radius fractures. Additionally, there was no difference in the conventional indications for surgery following the initial reduction between the two groups. These findings suggest that routine use of fluoroscopy in the ED for such fractures may need reconsideration. While fluoroscopy remains the gold standard, non-fluoroscopy-guided reduction could be a viable alternative.

## Introduction

Distal radius fractures are the most common type of fracture [1], accounting for 17.5% of all fractures in adults [2], with an annual incidence exceeding 640,000 in the US [3]. Most distal radius fractures are initially managed by closed reduction and casting with a plaster cast [1, 4]. Reduction is a crucial step in the definitive treatment of most displaced fractures, and the quality of the reduction, as shown in post-reduction radiographs, plays a significant role in determining the appropriate course of treatment. If optimal reduction is achieved, conservative treatment may be sufficient. However, if the reduction is inadequate, open reduction and internal fixation surgery may be necessary to restore the anatomic alignment of the fracture [5].

The use of fluoroscopy in the ED for the reduction of distal radius fractures has gained increasing popularity over the last two decades [6]. This trend is largely driven by the assumption that real-time X-ray visualization enhances radiographic outcomes and may decrease the need for surgical intervention. A critical limitation of traditional closed reduction methods is that they are essentially “blind” and guided solely by pre-reduction and post-reduction X-rays. As a result, the clinician may complete the reduction and casting, only to discover on post-reduction imaging that alignment remains inadequate, necessitating a repeat of the entire procedure. Fluoroscopy offers the significant advantage of continuous intra-procedural visualization, potentially reducing the number of reduction attempts, minimizing treatment time, bony and soft tissue manipulation, and improving the overall quality of fracture alignment [6].

Earlier studies have shown that fluoroscopy-assisted reduction, particularly in pediatric patients with distal radius and wrist fractures, results in improved radiographic outcomes and fewer reduction attempts [7]. However, fluoroscopy uses continuous X-ray beams to produce real-time video imaging, which leads to higher cumulative radiation exposure compared to standard static X-rays [8]. Radiation dose rates during fluoroscopy typically range between 20–60 mGy/min, and even a single second can deliver approximately 0.33–1 mGy depending on settings [9, 10]. In contrast, extremity X-rays usually involve much lower doses (e.g., ~ 1 µGy for a hand X-ray) [11]. While the doses during fluoroscopy generally remain below harmful thresholds if properly managed, this prolonged exposure still raises concerns about potential risks, including skin damage and long-term malignancy, particularly with repeated use [8, 12]. Moreover, individual fluoroscopic frames may lack the image resolution of conventional X-rays [13], which can hinder accurate assessment. These factors have contributed to recent discussions questioning the clinical benefit of fluoroscopy in closed fracture reductions. Current evidence suggests that fluoroscopy-assisted closed reductions of distal radius fractures in adults yield radiographic results comparable to those achieved without fluoroscopy [6, 14]. Additionally, data indicate that patients undergoing fluoroscopy-assisted reduction require more reduction attempts but achieve similar radiographic outcomes and a comparable rate of surgical intervention [14]. Interestingly, Orthopedic residents have reported that reductions felt more difficult when using fluoroscopy than without. Finally, some authors have questioned the value of routine ED reduction when surgery is already planned. In a randomized study, Löw et al. evaluated pain scores, median nerve injury, and clinical and radiographic outcomes, and found no disadvantage to omitting closed reduction prior to surgery. Nevertheless, reduction remains appropriate when conservative treatment is a realistic option [15].

The primary goal of this study is to assess the radiographic and clinical outcomes of adult distal radius fractures, with or without the use of fluoroscopy in an ED setting. The primary outcome measures include radiographic parameters on post-reduction radiographs.

## Methods

This study included electronic records and imaging data of adult patients (aged 18 and older) who sought medical attention for distal radius fractures requiring reduction. The inclusion criteria covered the period from January 1, 2015, to April 1, 2021, allowing for a diverse range of cases during this timeframe.

Two distinct patient groups were identified based on the use of fluoroscopy during the reduction procedure. The fluoroscopy-assisted reduction (FAR) group underwent reduction with intraoperative fluoroscopic guidance using a C-arm, enabling real-time evaluation and positional adjustments prior to final cast application. In contrast, the control group included patients who underwent reduction and casting without fluoroscopic assistance. These patients were treated between September 1, 2019, and March 4, 2020, during a period when fluoroscopy was not available at the medical center.

Exclusion criteria included: patients under the age of 18, those without adequate clinical or radiographic follow-up, open fractures, fractures that did not undergo reduction, patients who had received initial treatment at another medical center prior to arrival at the study facility, pathological fractures, fractures involving the ulna (excluding ulnar styloid fractures) that could affect relevant measurements, and fractures in patients with concomitant ipsilateral upper extremity fractures.

**Fig. 1:**
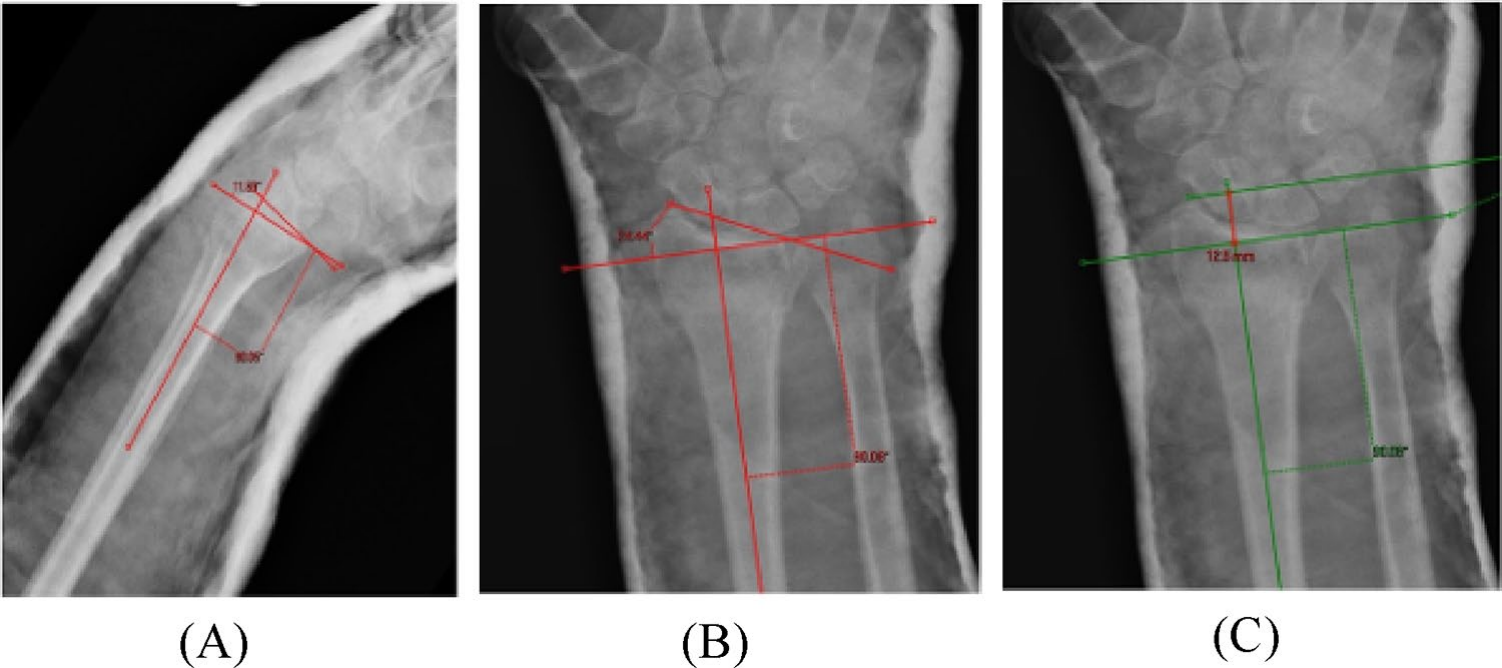
**A** Measurement of Dorsal Angulation on Lateral Radiograph; **B** Measurement of Wrist Inclination on PA Radiograph; **C** Measurement of Wrist Length on PA Radiograph;

Upon obtaining institutional review board approval, all patients who visited the level I trauma center during the study period and met the inclusion criteria were allocated to either the FAR or control group. Demographic data, comorbidities, referral date for ED care, length of ED stay, fracture classification, and pre- and post-reduction radiographic measurements (radial height, volar tilt, radial inclination, and articular step-of Fig. 1) were retrospectively collected for each patient.

Fractures were classified according to the AO classification, distinguishing between type 23-A (extra-articular) and 23-C (intra-articular) fractures [16]. Additionally, fracture stability was assessed using Lafontaine’s criteria [17]. Fractures exhibiting three or more positive parameters are considered unstable and at risk for loss of alignment, even with cast treatment.

The treatment course followed these steps: the patient's initial arrival to the ED, initial radiographs of the affected hand in posteroanterior (PA) and lateral views to identify the distal radius fracture, local anesthesia with a hematoma block, mechanical traction through ‘finger-traps’ [18], and manual reduction. Following reduction, a circumferential below-elbow cast was applied with a three-point fixation. Patients were instructed to elevate the hand, monitor pain and swelling, and seek medical attention if symptoms worsened. Routine follow-up was arranged. When clinically indicated, the cast was split to decompress and relieve swelling/pain, with cast replacement performed as needed. All patients underwent standard post-reduction PA and lateral wrist radiographs at the study center, regardless of intra-procedural fluoroscopy use, to confirm maintenance of the reduction and for documentation/measurements.

An initial closed reduction was performed on all fracture types, even when surgery was planned, to reduce pain and swelling, protect the soft tissues, and maintain provisional alignment while definitive management was arranged. Fractures meeting instability criteria (e.g., Lafontaine) were triaged for early outpatient review and/or operative intervention according to severity and the anticipated risk of failure in a cast. Patients who were indicated for surgery and proceeded to surgery were excluded from the follow-up alignment analysis. Among patients treated nonoperatively, follow-up radiographs were reviewed to confirm that alignment was maintained; cases requiring cast splitting were not classified as failures.

Although the Lafontaine and AO criteria help predict fracture stability, they do not, by themselves, mandate surgery. In this study, surgery was indicated for radial shortening > 3 mm, dorsal angulation > 10°, or intra-articular step/step off > 2 mm [5]. 

### Statistical analysis

Statistical analysis was performed using a combination of parametric and non-parametric tests, with non-parametric tests chosen based on the data distribution. Multiple imputation techniques were used to handle missing data, ensuring the integrity of the analysis. Effect sizes were calculated for key comparisons to assess the clinical significance of observed differences. Baseline characteristics, comorbidities, and outcomes were compared between the fluoroscopy and control groups. Differences in clinical and radiographic metrics were examined using the Chi-Square test and Fisher's exact test for categorical variables, and Student's t-test for continuous variables. The threshold for statistical significance was set at p = 0.05. All statistical analyses were conducted using SPSS Statistical Software (SPSS 24.0, IBM Inc., Somers, NY).

## Results

Eighty-five patients were included in the study with an average age of 58.9. The FAR group included 45 patients, and the control group included 40 patients. No significant differences were observed in average age and gender distribution between the two groups. The mean ages were 56.2 and 65 years in the FAR and control groups, respectively (p = 0.14). Similarly, no significant differences were observed in radiographic characteristics of fractures, including radial length, inclination, posterior angulation, and articular involvement. Additional demographic and radiographic baseline characteristics of the participants are presented in Table 1.

**Table 1:** Patient Demographics and Injury Characteristics

	Fluoroscopy	No Fluoroscopy	P-Value
	N = 45	N = 40	
Age (mean)	56.2 (SD 19.1)	62 (SD 16.7)	0.14
Gender			0.64
Male	16 (35.5%)	12 (30%)	
Female	29 (64.5%)	28 (70%)	
Lafontaine			0.17
Stable	12 (26.7%)	17 (42.5%)	
Unstable	33 (73.3%)	23 (57.5%)	
AO (23A,C)*			0.004
A2	6	8	
A3	22	11	
C1	1	12	
C2	13	8	
C3	3	1	
Radial Length (mm)	5.92 (SD 4.85)	6.7 (SD 5.16)	0.43
Inclination (degrees)	15.5 (SD 6.12)	17 (SD 6.8)	0.29
Angulation (degrees)	24.7 (SD 11.12)	21.75 (SD 11.7)	0.23
Step Off (mm)	0.05 (SD 0.26)	0.36 (SD 0.78)	0.014
Dorsal Comminution n, (%)	36 (80%)	23 (57.5%)	0.03
Ulnar Styloid Fracture, n (%)	28 (62.2%)	19 (47.5%)	0.19
Surgical treatment	13 (28.8%)	11 (27.5%)	0.99

*The table includes only the AO-classified fractures that were included in the study

When comparing AO classification between the groups, no significant difference was noted between type 23-A (extra-articular fractures) and type 23-C (intra-articular fractures).

A statistically significant difference was observed in the posterior angulation of the fractures between the two groups (FAR group: 80%, control group: 57%, p = 0.03). However, no significant difference was found in fracture stability according to Lafontaine’s criteria [17] (p = 0.17), which includes posterior angulation as one of its variables.

Analysis and comparison of post-reduction and casting radiographs revealed no significant differences between the two groups in conventional radiographic measurements (p > 0.48), including radial length, inclination, posterior angulation, and articular grade, as shown in Table 2**.**

**Table 2:** Radiographic Outcomes

	Fluoroscopy	No fluoroscopy	P-value
	N = 45	N = 40	
Radial Length (mean, mm)	10.8 (SD 3.5)	11 (SD 3.16)	0.8
Inclination (mean, mm)	20.6 (SD 4.3)	21.26 (SD 4)	0.46
Angulation (mean, degrees)	4.2 (SD 6.6)	3.4 (SD 7.14)	0.59
Step off (mean, mm)	0.14 (SD 0.36)	0.28 (SD 0.68)	0.48

Clinically, no significant difference was observed between the two groups regarding the indication for surgery. As stated above, surgical indications were based on post-reduction and casting X-rays, following standard parameters such as radial shortening of more than 3 mm, posterior angulation of more than 10 degrees, and articular involvement of more than 2 mm [5]. Furthermore, no significant difference was found in the number of patients who underwent surgery by the end of the study. Among patients managed conservatively, follow-up radiographs demonstrated maintenance of alignment without conversion to surgery.

Additional variables examined included the duration of hospital stay from admission to discharge. In the FAR group, the average time spent in the ED was 171.6 min, compared to 185.1 min in the control group, with no statistically significant difference (p = 0.63). Within one week of initial treatment, 9/45 (20.0%) patients in the FAR group and 6/40 (15.0%) in the non-fluoroscopy group returned to the ED for cast-related complications (swelling/pressure) and underwent cast splitting for relief; the difference was not statistically significant (p = 0.58**)**, indicating no clear advantage of FAR in reducing early cast issues.

## Discussion

This study evaluated whether the use of fluoroscopy during closed reduction of distal radius fractures improves radiographic outcomes, with a particular focus on its added clinical value. Fluoroscopy provides dynamic, real-time imaging that allows for intra-procedural adjustments, potentially enhancing reduction accuracy [7]. However, this imaging modality introduces variability and subjectivity, and its lower image resolution compared to static X-rays may limit its utility for definitive assessment [13]. Notably, all final measurements and assessments in this study were made from plain radiographs, not fluoroscopic images, underscoring fluoroscopy’s limitations for accurate, standardized documentation. Because post-reduction radiographs are recommended for diagnostic documentation and measurement [19, 20], all patients received standard post-reduction PA and lateral wrist radiographs, regardless of intra-procedural fluoroscopy use. These radiographs also served to confirm maintenance of alignment as swelling subsided and other deforming forces could act on the fracture. Moreover, because definitive operative scheduling could be delayed, an initial closed reduction was used to mitigate pain and swelling, protect soft tissues, and maintain provisional alignment pending surgery. This approach was particularly relevant for fractures meeting instability criteria (e.g., Lafontaine), which were prioritized for early clinic review and operative management when indicated.

Radiation exposure is another important consideration. Fluoroscopy, due to its continuous X-ray beam, generally delivers higher cumulative doses than conventional X-rays [8]. For example, a single second of fluoroscopy can deliver approximately 0.33–1 mGy, whereas a standard extremity X-ray typically delivers only ~ 1 µGy [9–11]. This discrepancy underscores the importance of balancing the procedural benefits of real-time imaging with the potential long-term risks of cumulative radiation, particularly in younger or more vulnerable populations.

Cost-related factors further limit fluoroscopy's widespread implementation. In addition to the substantial expense of acquiring a fluoroscopy unit, there are ancillary costs related to installation and operation, such as constructing lead-lined or non-penetrable walls and providing appropriate protective gear for staff. These infrastructure and safety requirements may limit the widespread use of fluoroscopy in some settings.

Overall, while fluoroscopy offers certain procedural advantages, its limitations in accuracy, radiation exposure, and cost must be carefully considered. As highlighted previously, plain radiographs remain the standard for assessing fracture alignment and serve as the reference for all measurements in this study.

Prior studies have reported mixed findings. In pediatric populations, some evidence suggests improved radiographic outcomes and lower re-reduction rates with fluoroscopy [7], but in adults, fluoroscopy has not consistently demonstrated a significant impact on radiographic or clinical outcomes following closed reduction [14, 16].

This study focused on adult patients with distal radius fractures characterized by angulation and shortening. Fracture types such as Barton fractures, AO Type B patterns, and those involving the ulna were excluded to allow more precise measurement of key radiographic variables: dorsal tilt, shortening, angulation, and intra-articular involvement.

The study found no significant differences between the FAR and control groups in post-reduction radiographic outcomes or in the proportion meeting surgical indications. Return visits for cast splitting due to pain/swelling were not significantly different either; however, more patients in the FAR group returned for cast splitting, indicating no advantage for FAR.

While the average ED stay was shorter in the FAR group (171.6 min vs. 185.1 min), the 13.5-min reduction was not statistically significant. These findings are consistent with previous reports, such as Dailey et al. [14], who also observed a non-significant reduction in ED stay duration with fluoroscopy. Some clinicians in the study reported that using fluoroscopy made the reduction subjectively more challenging, possibly due to the need to interpret dynamic rather than static images.

Finally, although not statistically significant, there was a trend toward more return visits within the first week in the fluoroscopy group due to cast-related pressure discomfort (9 vs. 6 patients). This observation, while preliminary, may have important clinical and economic implications and warrants further investigation in larger cohorts.

In summary, while fluoroscopy may provide precision in fracture alignment, it comes with significant radiation exposure and associated costs. The findings suggest that non-fluoroscopy-guided reduction can be an effective alternative in settings where fluoroscopy is not available or contraindicated, without compromising key clinical outcomes such as functional recovery or fracture healing. These results are consistent with recent literature that questions the necessity of routine fluoroscopy in closed reductions of distal radius fractures. Although this study showed significant differences in posterior angulation and radial height between the fluoroscopy-guided and non-fluoroscopy-guided groups, these differences, while statistically significant, did not translate into meaningful clinical benefits, such as improved functional recovery or reduced surgery rates. This suggests that while fluoroscopy may offer more precise alignment, the clinical advantages of this added precision are marginal.

### Limitations

This study has several limitations that should be considered. First, it is a single-center, retrospective observational study with a relatively small sample size, which may limit the generalizability of the findings. While the sample size is limited, the study still provides valuable insights into the comparison of fluoroscopy and non-fluoroscopy-guided reduction techniques. Additionally, although the reduction and casting technique was standardized, the procedures were performed by different specialists at various stages of their training, which could introduce some variability. However, this reflects real-world clinical practice, where multiple providers may be involved in patient care. Furthermore, there is a possibility that some patients chose not to be treated at the study institution or opted for care at other facilities, which might have introduced selection bias. Also, management pathways were influenced by potential delays in operative scheduling, which may have prompted initial closed reduction to control symptoms and protect soft tissues while definitive treatment was arranged. Finally, the study focused on time-zero comparisons; follow-up assessments were descriptive and limited to patients managed non-operatively.

## Conclusion

This study aimed to assess the impact of fluoroscopy during the reduction of distal radius fractures. The findings indicate that fluoroscopy-assisted reduction does not provide a statistically significant advantage over non-fluoroscopy-assisted reduction in terms of radiographic characteristics, including radius length, inclination, posterior angulation, and articular involvement. Additionally, there was no difference in surgical indications or post-reduction outcomes, and the use of fluoroscopy did not significantly reduce the time spent in the ED or the rate of return visits due to plaster complications. Given these results, the routine use of fluoroscopy in the ED for distal radius fractures may not be necessary. While fluoroscopy offers higher precision in fracture alignment, it has some disadvantages, such as increased radiation exposure and associated costs. These findings suggest that the benefits of fluoroscopy should be reconsidered, especially when non-fluoroscopy-guided reduction can achieve comparable outcomes. While fluoroscopy remains the gold standard for reduction, non-fluoroscopy-guided reduction may serve as a viable alternative in certain emergency settings.

## Data Availability

No datasets were generated or analysed during the current study.
